# Aberrant Notch-signaling promotes tumor angiogenesis in esophageal squamous-cell carcinoma

**DOI:** 10.1038/s41392-025-02309-5

**Published:** 2025-07-22

**Authors:** Cainan Li, Pujie Wu, Xiaoting Xie, Xinjie Chen, Liping Chen, Liang Zhu, Zhixuan Xuan, Tianyuan Liu, Wen Tan, Shaosen Zhang, Dongxin Lin, Chen Wu

**Affiliations:** 1 Department of Etiology and Carcinogenesis, National Cancer Center/National Clinical Research Center for Cancer/Cancer Hospital, Chinese Academy of Medical Sciences and Peking Union Medical College, Beijing 100021, China; 2 Changping Laboratory, Chinese Academy of Medical Sciences and Peking Union Medical College, Beijing 102206, China; 3 Key Laboratory of Cancer Genomic Biology, Chinese Academy of Medical Sciences and Peking Union Medical College, Beijing 100021, China; 4 Collaborative Innovation Center for Cancer Personalized Medicine, Nanjing Medical University, Nanjing 211166, China; 5 Sun Yat-sen University Cancer Center, State Key Laboratory of Oncology in South China, Guangzhou 510060, China; 6 CAMS Oxford Institute, Chinese Academy of Medical Sciences, Beijing 100006, China

**Keywords:** Tumour angiogenesis, Gastrointestinal cancer

## Abstract

Esophageal squamous-cell carcinoma (ESCC) is one of the most common gastrointestinal cancers in China, characterized by high malignancy and poor prognosis. Nowadays, the therapeutic options for this cancer are very limited. Notch-signaling is often overactivated in ESCC, but its role remains to be fully elucidated. Here, we demonstrate that aberrant Notch-signaling plays an important role in tumor angiogenesis. In clinical ESCC samples, Notch-signaling activation scores were significantly correlated with tumor microvascular density, advanced TNM stages, and short patient survival time. Silencing Notch-signaling substantially suppressed the ability of ESCC cells to promote angiogenesis in vitro and in vivo. By integrating analysis of CUT&Tag and RNA sequencing data, we identified ubiquitin-specific protease 5 (USP5) as a Notch-signaling downstream effector that is transcriptionally upregulated by the NOTCH1 intracellular domain (NICD1)–RBPJ complex and mediates tumor angiogenesis. USP5 stabilized STAT3 via its deubiquitination function, thereby enhancing the production of pro-angiogenic factors by cancer cells, including VEGF, ANGPT2, and CXCL1. We showed that chemotherapy combined with the USP5 inhibitor can additionally repress tumor growth and angiogenesis in mice. These findings explain why ESCC cells have much fewer *NOTCH1* mutations than normal and precancerous epithelium, reveal a novel mechanism for Notch-signaling to drive tumor angiogenesis via the NOTCH1–USP5–STAT3 axis, and open a potential new avenue for anti-tumor angiogenesis therapy.

## Introduction

Esophageal squamous-cell carcinoma (ESCC) is one of the most aggressive and deadly cancers, with a particularly high incidence in East Asia.^1^ The disease is frequently diagnosed at advanced stages, leading to poor prognosis due to its late detection, high recurrence rate, and resistance to conventional treatments.^2,3^ Despite significant advancements in treatment strategies, such as chemotherapy and radiotherapy,^4,5^ the survival rate of ESCC patients remains low^6^ underscoring the need for a deeper understanding of its molecular drivers. Although various genetic^7,8^ and epigenetic^9^ alterations have been implicated in ESCC tumorigenesis, the precise molecular mechanisms underlying its initiation and progression remain incompletely understood.

Multiple large-scale genomic sequencing studies have identified *NOTCH1* as a frequently mutated gene in ESCC (nearly 20%), second only to *TP53* mutations.^7,8,10–13^ Interestingly, the mutation frequency of *NOTCH1* is even higher in normal aged esophageal epithelium.^14,15^ *NOTCH1* mutations seem to exhibit a dual functionality; in normal aged esophageal epithelium, they drive clonal expansion, whereas in ESCC, they suppress tumor growth.^16,17^ The Notch signaling pathway operates through the transmembrane receptor NOTCH1, which binds ligands from the Jagged (Jag) families and the Delta-like (Dll) families. Upon ligand binding, NOTCH1 undergoes proteolytic cleavage by ADAM and γ-secretase, releasing the NOTCH1 intracellular domain (NICD1). NICD1 then translocates to the nucleus, where it interacts with the RBPJ transcriptional complex, recruiting co-activators such as MAML, converting it from a repressive to an active state. This activation triggers the transcription of downstream target genes, including *HES1*, *HES4*, *HEY1*, and *MYC*.^18,19^ Mutations in *NOTCH1* are mainly concentrated in the extracellular EGF-like domain, impairing ligand binding and leading to the loss of Notch signaling function.^15^ Although *NOTCH1* mutations occur in nearly 20% of ESCC cases, a larger proportion of patients without mutations still exhibit functional Notch signaling in their tumors. Moreover, in contrast to normal tissue, Notch signaling is significantly overactivated in tumor tissues. Activation of Notch signaling has been reported to be associated with various biological processes such as stemness, metastasis, and epithelial-to-mesenchymal transition (EMT)^20–22^ in multiple types of human cancer. However, the functions and the underlying mechanisms of Notch signaling activation in ESCC remain poorly understood.

Angiogenesis, the process of new blood vessel formation from existing vasculature, is a hallmark of cancer and plays a critical role in tumor progression and metastasis. Tumors require an increased supply of oxygen and nutrients to sustain their rapid growth, and in response, they secrete pro-angiogenic factors that promote neovascularization in the tumor microenvironment (TME).^23^ In ESCC, angiogenesis is particularly pronounced and correlates with tumor aggressiveness, advanced stages, and poor prognosis. The tumor vasculature is often disorganized, with leaky blood vessels that are inefficient at providing adequate oxygen, contributing to hypoxic regions within the tumor. In turn, hypoxia further stimulates angiogenic signaling, creating a feedback loop that drives tumor progression. Common pro-angiogenic factors include vascular endothelial growth factor (VEGF),^24^ fibroblast growth factors (FGFs),^25^ and angiopoietin-2 (ANGPT2),^26^ which promote endothelial cell migration, proliferation, and tube formation.^27^ Although anti-angiogenic therapies have been developed, their effectiveness is often limited due to compensatory mechanisms and resistance, highlighting the need for novel therapeutic strategies targeting angiogenesis in ESCC.

In the present study, we have explored the relationship between Notch signaling and angiogenesis in ESCC by reanalyzing our previously published single-cell RNA sequencing dataset.^28^ We have found that high Notch signaling pathway scores in epithelial cells are significantly associated with increased angiogenesis in tumors. Furthermore, immunofluorescence staining of NICD1, a marker of Notch signaling activation, and CD31, an endothelial cell marker, has shown that ESCCs with high NICD1 expression had increased microvascular density. Functionally, knockout of *NOTCH1* in ESCC cells inhibits endothelial cell migration and tube formation in vitro, reduces microvascular density in subcutaneous tumor xenografts in vivo, and decreases microvascular density in carcinogen-induced ESCC among *Notch1*-knockout mice. Mechanistically, we have identified for the first time that Notch signaling activation, through the NICD1–RBPJ complex, transcriptionally upregulates USP5, a deubiquitinating enzyme that stabilizes STAT3 by preventing its ubiquitination and proteasome-mediated degradation. Stabilized STAT3 then promotes the expression and secretion of pro-angiogenic factors such as VEGF, ANGPT2, and CXCL1, which further enhance endothelial cell function and angiogenesis. Our results also demonstrate that inhibiting the Notch/USP5 signaling significantly reduces the microvascular density and tumor volume in mice and significantly increases the tumor suppressive effect of chemotherapy for ESCC. Together, these findings suggest that the Notch signaling pathway plays a crucial role in promoting angiogenesis in ESCC, providing a new insight into the molecular mechanism driving tumor progression and potential therapeutic targets for ESCC.

## Results

### Aberrant Notch-signaling promotes angiogenesis and correlates with poor prognosis in ESCC

**Fig. 1:**
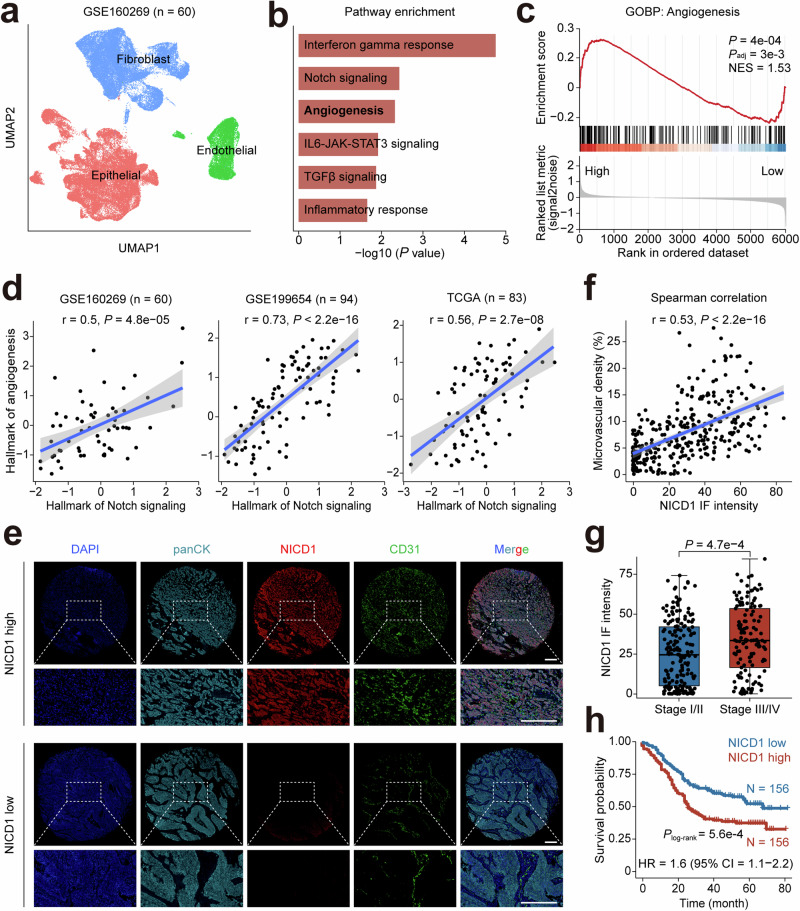
Aberrant Notch-signaling activation promotes angiogenesis and correlates with poor prognosis in ESCC. **a** UMAP plot of 86,933 single cells from ESCC tumor tissues, illustrating the distribution of three distinct cell types, i.e., epithelial cells, endothelial cells and fibroblasts. **b** Pathway enrichment analysis of differentially expressed genes between Notch-signaling score high and Notch-signaling score low groups. **c** Gene set enrichment analysis between Notch-signaling score high and Notch-signaling score low groups shows significantly enriched angiogenesis in the Notch-signaling score high group. **d** Spearman correlations between the Notch-signaling scores and the angiogenesis scores in three different datasets. The shaded areas represent 95% confidence intervals. **e**, **f** Significant positive correlation between NICD1 and CD31 protein levels in tissue microarrays consisting of 312 ESCC samples. Panel (**e**) shows multiplexed immunofluorescence staining images and panel (**f**) shows Spearman correlation between NICD1 and CD31 levels, with the shaded area representing 95% confidence interval. Epithelial cells were stained with panCK, and nuclei were stained with DAPI. Scale bar, 300 μm. **g** Boxplot showing significantly different NICD1 immunofluorescence intensities in early ESCC (TNM stage I/II) or advanced ESCC (TNM stage III/IV). Data are median (central line) with the 25−75% interquartile range, and whiskers represent ±1.5 times the interquartile range. *P* value was derived from the Wilcoxon rank-sum test. **h** Kaplan–Meier survival analysis of 312 ESCC patients stratified by median NICD1 immunofluorescence (IF) intensity. *P* value was derived from the log-rank test. The hazard ratio (HR) and 95% confidence interval (CI) were calculated using a multivariate Cox proportional hazard model, adjusting for age, sex, tumor stage, smoking, and drinking status

We first analyzed our previously published single-cell RNA sequencing dataset that consisted of 60 ESCC patients.^28^ Based on the Notch signaling pathway scores in epithelial cells, we divided these ESCCs into high score or low score groups (Fig. 1a and Supplementary Fig. 1a). Pathway enrichment analysis of differentially expressed genes between these two groups revealed significant enrichment of high score group ESCCs in pathways such as interferon gamma response, Notch signaling and angiogenesis (Fig. 1b). Gene set enrichment analysis also indicated that the angiogenesis pathway was significantly enriched in Notch signaling score high ESCCs (Fig. 1c). In the single-cell RNA sequencing dataset of ESCC, the Notch signaling pathway scores in epithelial cells were significantly and positively correlated with the angiogenesis pathway score, and this positive correlation was also seen in other two bulk RNA sequencing datasets, i.e., GSE199654^7^ and TCGA-ESCA (Fig. 1d).

We then performed multiplexed immunofluorescence analysis on a tissue array consisting of 312 ESCC tumors. NICD1 staining intensity served as a marker for Notch signaling activation, and an anti-CD31 antibody was used to stain vascular endothelial cells for the quantification of microvascular density in the tissue (Fig. 1e). The results showed a significant positive correlation between the levels of NICD1 fluorescence intensity and CD31 density (Fig. 1f). Moreover, stratification analysis showed that the level of Notch signaling activation, indicated by NICD1 staining intensity, was significantly higher in advanced ESCC (TNM stage III/IV) than in early staged ESCC (TNM stage I/II) (Fig. 1g), and significantly higher in invasive tumors and tumors having lymph node metastasis compared with non-invasive tumors and tumors without lymph node metastasis (Supplementary Fig. 1b, c and Supplementary Table 1). More significantly, we found that patients with high NICD1 staining intensity had significantly shorter survival time than those with low NICD1 staining levels (median survival time 26 months versus 68 months, log-rank *P* = 5.6e−4). The hazard ratio for death of high NICD1 staining intensity, adjusted for age, sex, tumor stage, smoking, and drinking status, was 1.6 (95% confidence interval = 1.1−2.2; Fig. 1h). Collectively, these results indicate that high activation of the Notch signaling pathway in ESCC may promote tumor progression via angiogenesis.

### Aberrant Notch-signaling activation promotes ESCC tumor angiogenesis

**Fig. 2:**
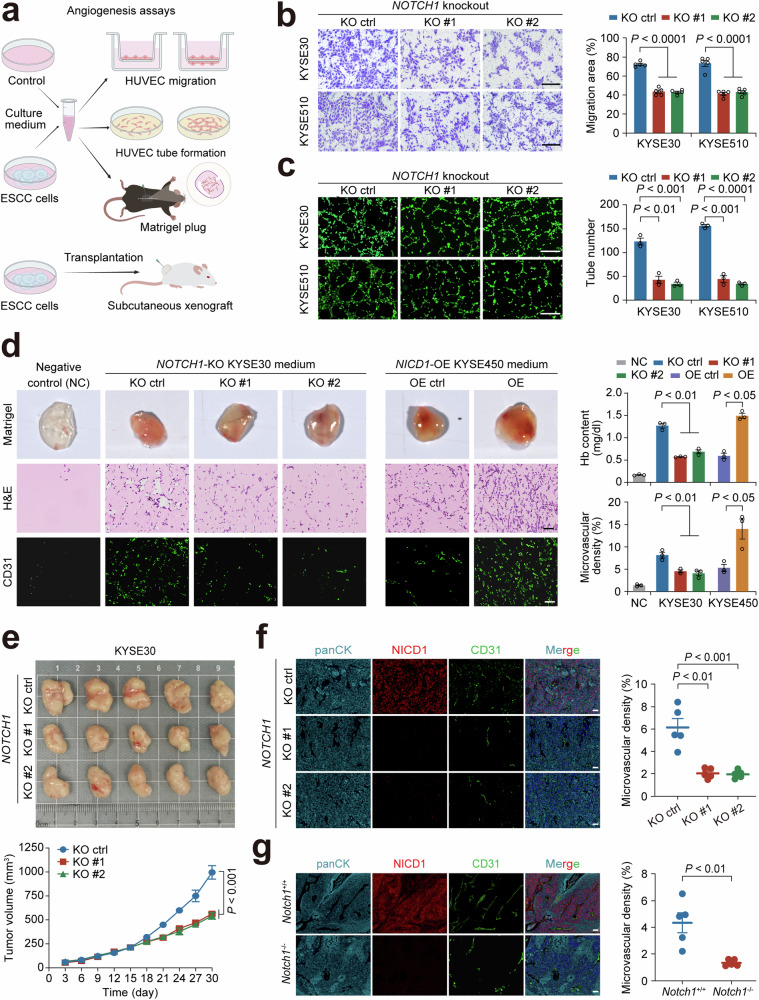
Aberrant Notch-signaling activation promotes tumor angiogenesis. **a** The schematics of in vitro and in vivo experiments to evaluate the effect of Notch signaling on angiogenesis. HUVEC, human umbilical vein endothelial cell. **b** The effect of the *NOTCH1*-knocked out ESCC cell culture medium on HUVEC migration. The left panel shows images of HUVECs in Transwell assays, and the right panel shows the quantitative statistics. Scale bar, 300 μm. **c** The effect of *NOTCH1*-knocked out ESCC cell culture medium on HUVEC tube formation. The left panel shows images of the tube density, and the right panel shows the quantitative statistics. Scale bar, 500 μm. **d** The effect of *NOTCH1*-knocked out or *NICD1*-overexpressed ESCC cell culture medium on hemoglobin (Hb) content and microvascular density in the Matrigel plug assays. The left panel shows Matrigel plug images and sections with H&E or CD31 staining, and the right panel shows the quantitative statistics. Scale bar, 50 μm. **e** *NOTCH1* knockout substantially reduced the growth rate of mouse xenografts derived from human ESCC cells. The upper panel shows xenograft tumors derived from *NOTCH1*-knockout or *NOTCH1*-non-knockout ESCC cells at the end of the experiment, and the lower panel shows the different growth curves of xenografts in mice during the experiment. **f** *NOTCH1*-knockout ESCC cell-derived xenografts had significantly reduced microvascular density compared with *NOTCH1*-non-knockout ESCC cell-derived xenografts. The left panel shows representative immunofluorescence images of NICD1 and CD31, and the right panel shows quantitative statistics. The ESCC xenograft tumor tissues were identified by panCK staining. Scale bar, 50 μm. **g** NICD1 and CD31 in 4-NQO-induced ESCC in *Notch1*^*+/+*^ or *Notch1*^−/−^ mice. The left panel shows immunofluorescence staining images of NICD1 and CD31, and the right panel shows the quantitative statistics. The 4-NQO-induced ESCC was identified by panCK staining. Scale bar, 50 μm. Data are mean ± SEM from 5 (**b**) and 3 (**c**) independent experiments, and each had 3 replicates, or 3 (**d**) and 5 (**e**–**g**) mice. *P* values were derived from Student’s *t*-test

We next performed several experiments to explore whether Notch signaling is involved in ESCC tumor angiogenesis. First, we silenced *NOTCH1* expression in KYSE30 and KYSE510 cell lines originally having high NICD1 expression, and overexpressed *NICD1* in KYSE450 and KYSE150 cell lines originally having no NICD1 expression (Supplementary Fig. 2a and b). These cell lines with the gain or loss of NICD1 function were used to examine the effect of Notch signaling on angiogenesis in vitro. Conditioned medium from these cell cultures was collected and applied to HUVEC migration and tube formation assays (Fig. 2a). The results showed that compared with control medium, medium from ESCC cells with loss of NOTCH1 function significantly inhibited the HUVEC migration and tube formation capacities (Fig. 2b, c and Supplementary Fig. 2c). In contrast, the medium from ESCC cells with gain of NICD1 function significantly enhanced HUVEC migration and tube formation abilities (Supplementary Fig. 2d and e). Second, we performed Matrigel plug assays and generated subcutaneous xenografts in mice to evaluate the role of Notch signaling in angiogenesis in vivo (Fig. 2a). We found that in Matrigel plug assays, the conditioned medium from the culture of ESCC cells without NOTCH1 expression suppressed the pro-angiogenic capacity as indicated by decreased hemoglobin content and microvascular density; however, the conditioned medium from the culture of ESCC cells with NICD1 expression significantly enhanced this angiogenic phenotypes, suggesting a critical pro-angiogenic role of Notch signaling in ESCC (Fig. 2d). In mouse xenografts derived from human ESCC cells, we found that *NOTCH1-*knockout tumors had substantially suppressed growth rate compared with *NOTCH1-*non-knockout tumors (Fig. 2e). Multiplexed immunofluorescence staining analysis of tumor sections revealed that *NOTCH1*-knockout tumors had significantly reduced microvascular density compared with *NOTCH1-*non-knockout tumors (Fig. 2f). Both in vitro CCK-8 assays for cell proliferation and Ki-67 staining of in vivo xenograft tumors demonstrated that the tumor-suppressive effect of *NOTCH1* knockout was not attributable to the effect on cell proliferation (Supplementary Fig. 2f and g). In addition, based on the 4-Nitroquinoline 1-oxide (4-NQO)-induced primary ESCC model in esophageal epithelium-specific *Notch1* knockout mice as described in our previous study,^29^ we also analyzed the difference in the microvascular density in 4-NQO-induced primary ESCC between *Notch1*^*+/+*^ and *Notch1*^*−/−*^ mice, and the results showed that the microvascular density was significantly less in *Notch1*^*−/−*^ tumors than that in *Notch1*^*+/+*^ tumors (Fig. 2g). Together, these in vitro and in vivo findings indicate that Notch signaling activation enhances tumor angiogenesis in ESCC.

Given that Notch and hypoxia-inducible factor (HIF) signaling interact in various tumors and HIF is a key promoter of angiogenesis,^30–33^ we investigated whether Notch signaling-mediated angiogenesis in ESCC is driven by the HIF pathway. Gene set enrichment analysis revealed that ESCCs with high Notch signaling scores did not exhibit enrichment of the HIF1 signaling pathway, and there was no significant correlation between Notch signaling and HIF1 signaling scores (Supplementary Fig. 3a and b). Next, we examined the effects of *NOTCH1* knockout on key HIF pathway components in ESCC cells and found that the expression levels of HIF1α, HIF2α and HIF1β remained unchanged (Supplementary Fig. 3c). Similarly, siRNA-mediated *HIF1α* or *HIF2α* knockdown did not alter the activation level of Notch signaling (Supplementary Fig. 3d). Importantly, in vitro HUVEC migration and tube formation assays showed that *HIF1α* or *HIF2α* knockdown did not abrogate the pro-angiogenic phenotype induced by *NICD1* overexpression (Supplementary Fig. 3e−g). Together, these results suggest that Notch signaling activation promotes angiogenesis in ESCC in an HIF-independent manner.

### NICD1-RBPJ complex regulates *USP5* transcriptional expression

**Fig. 3:**
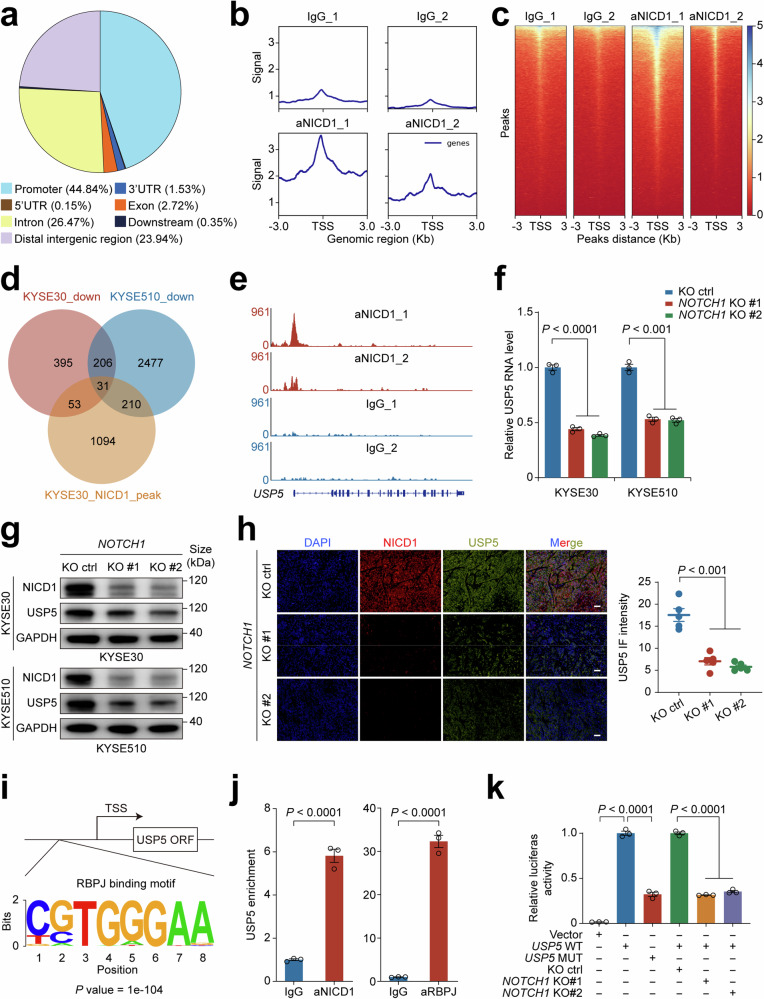
NICD1–RBPJ complex regulates USP5 transcriptional expression. **a** Pie plot showing the genomic distribution of NICD1 CUT&Tag peaks in KYSE30 cells. **b** NICD1 CUT&Tag signal height and position relative to the TSS for all genes in KYSE30 cells. **c** CUT&Tag density heatmap of NICD1 enrichment within 3 kb around TSS in KYSE30 cells. **d** Venn diagram showing the intersection results of NICD1 binding sites and protein-coding genes that had decreased mRNA levels due to *NOTCH1* knockout. **e** IGV tracks for *USP5* from NICD1 CUT&Tag analysis, showing NICD1 is enriched in the *USP5* promoter region. **f** *NOTCH1*-knockout cells had significantly reduced *USP5* RNA levels compared with control cells. *USP5* RNA levels were determined by qRT-PCR and normalized by *GAPDH* RNA levels. Data are mean ± SEM from three biological replicates. **g** Western blot analysis shows substantially decreased USP5 levels in *NOTCH1*-knockout ESCC cells compared with control cells. The experiment had three biological replicates. **h** *NOTCH1*-knockout ESCC cell-derived mouse xenografts had significantly reduced NICD1 and USP5 protein levels compared to control ESCC cell-derived xenografts. The left panel shows representative NICD1 and USP5 immunofluorescence staining images in tumor sections, and the right panel shows the quantitative statistics. Scale bar, 50 μm. Data are mean ± SEM from 5 mice. **i** In silico analysis indicates a potential RBPJ-binding site in the *USP5* promoter region. **j** Chromatin immunoprecipitation with anti-NICD1 or anti-RBPJ antibody-coupled qPCR assays demonstrates the binding of NICD1/RBPJ to the *USP5* promoter region in KYSE30 cells. Data are mean ± SEM from three independent experiments. **k** The results of reporter gene assays with plasmid constructs consisting of wild-type (WT) or mutant-type (MUT) *USP5* promoter in *NOTCH1*-knocked out or *NOTCH1*-nonknocked out KYSE30 cells. Data are mean ± SEM from 3 independent experiments. *P* values in this figure were derived from Student’s *t*-test

With the results described above, we next wanted to seek the mechanism underlying the pro-angiogenic function of Notch signaling. Since the canonical function of Notch signaling is to regulate gene transcription,^18,19^ we thus performed CUT&Tag coupled with next-generation sequencing using anti-NICD1 antibodies to visualize the NICD1 distribution in the genome. As a result, we identified 2067 NICD1-binding sites, among which 44.84% were located near the transcription start site (TSS) (Fig. 3a−c), including its well-known target genes such as *HES1* and *HES4* (Supplementary Fig. 4a). We also performed RNA sequencing on KYSE30 and KYSE510 cells to validate the effects of *NOTCH1* knockout on its target gene regulation. Integrative analysis of the CUT&Tag and RNA data revealed that there were 31 protein-coding genes presented NICD1-binding signals in their promoters and decreased mRNA levels when *NOTCH1* was knocked out (Fig. 3d). Notably, we found that among these 31 genes, the *USP5* locus encoding ubiquitin-specific protease 5, had the CUT&Tag peak signal second only to *HES1*, a well-known target gene of NICD1 (Fig. 3e and Supplementary Table 2). We performed immunofluorescence staining on the previously described ESCC tissue arrays and observed a significant positive correlation between NICD1 intensity and USP5 intensity (Supplementary Fig. 4b and c). Collectively, these findings strongly suggest that *USP5* may also be regulated by NICD1.

We further looked at the effect of loss or gain of *NOTCH1* gene function on USP5 expression in ESCC cells and found that knockout of *NOTCH1* gene significantly suppressed while overexpression of *NICD1* significantly enhanced USP5 expression at both RNA and protein levels (Fig. 3f−h, Supplementary Fig. 4d and e). Likewise, in the 4-NQO-induced primary ESCC mouse model, USP5 expression was also reduced in tumors from *Notch1*^−/−^ mice compared with those from *Notch1*^+/+^ mice (Supplementary Fig. 4f). Indeed, in silico analysis revealed a RBPJ binding motif in the promoter sequence of *USP5* (Fig. 3i). We thus conducted chromatin immunoprecipitation coupled qPCR assays and the results confirmed an interaction between NICD1/RBPJ and the *USP5* promoter (Fig. 3j and Supplementary Fig. 4g). We also performed reporter gene assays using the plasmid construct consisting of the wild-type or mutant-type *USP5* promoter sequence containing the putative RBPJ binding site. The results indicated that the plasmid containing wild-type *USP5* promoter had significantly higher luciferase activity compared with the plasmid containing mutant-type counterparts. Furthermore, the luciferase activity driven by the wild-type *USP5* promoter was significantly suppressed by *NOTCH1* knockout in cells (Fig. 3k and Supplementary Fig. 4h). These results support that *USP5* is a gene whose expression is controlled by NOTCH1 activation.

### Elevated USP5 mediates Notch signaling activation-induced tumor angiogenesis

**Fig. 4:**
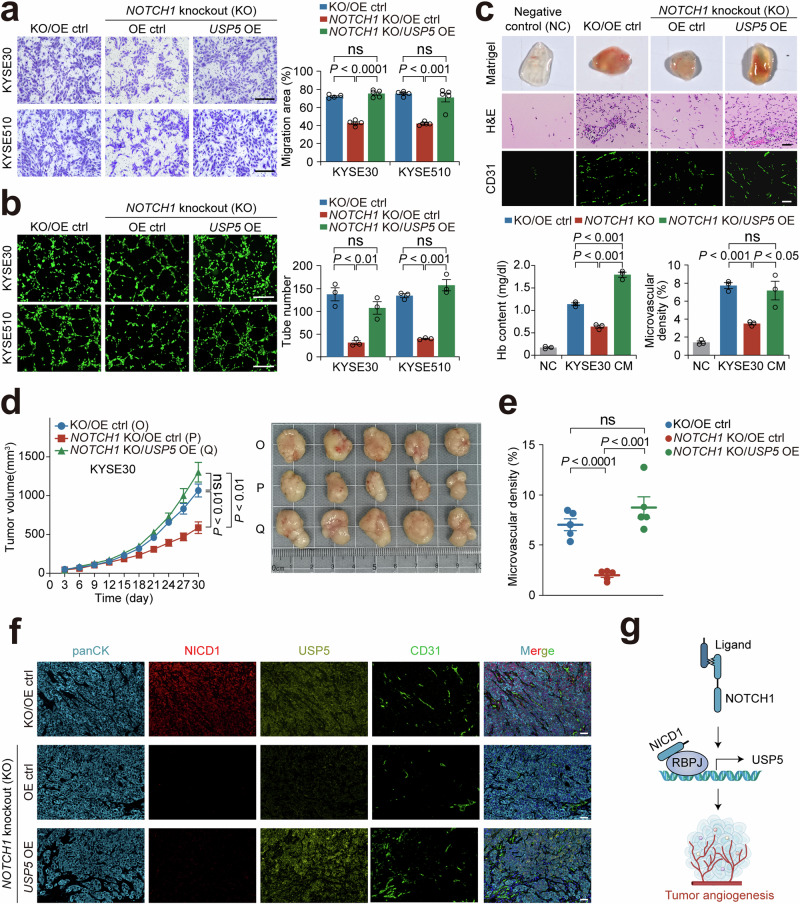
Elevated USP5 mediates Notch-signaling activation-induced tumor angiogenesis. **a** Ectopic overexpression of *USP5* in *NOTCH1*-knocked out ESCC cells can rescue the ability of ESCC cells to promote HUVEC migration. The left panel shows the Transwell assay images, and the right panel is the quantitative statistics. Scale bar, 300 μm. **b** Ectopic overexpression of *USP5* in *NOTCH1*-knocked out ESCC cells can rescue the ability of ESCC cells to promote HUVEC tube formation. The left panel shows the HUVEC tube density, and the right panel shows the quantitative statistics. Scale bar, 500 μm. **c** Ectopic overexpression of *USP5* in *NOTCH1*-knocked out ESCC cells can rescue the ability of ESCC cells to increase the hemoglobin (Hb) content and microvascular density in vivo in Matrigel plug assays. The upper panel shows Matrigel plug images and H&E or CD31 staining of their sections. The lower panel is the quantitative statistics. Scale bar, 50 μm. **d** Ectopic overexpression of *USP5* in *NOTCH1*-knocked out ESCC cells can restore the faster growth of ESCC cells-derived xenografts in mice. The left panel shows the xenograft growth curves during the experiment, and the right panel shows ESCC xenografts at the end of the experiment. **e**, **f** Ectopic overexpression of *USP5* in *NOTCH1*-knocked out ESCC cells can rescue the microvascular density in xenografts. Panel (**e**) shows the quantitative statistics, and panel (**f**) shows the images of NICD1, USP5 and CD31 immunofluorescence staining. The ESCC xenograft tumor tissues were identified by panCK staining. Scale bar, 50 μm. **g** A possible molecular mechanism for Notch-signaling to regulate angiogenesis in ESCC: via activating USP5 transcription. Data are mean ± SEM from 5 (**a**) and 3 (**b**) independent experiments and each had three replicates, or 3 (**c**) and 5 (**d, e**) mice. *P* values were derived from Student’s *t*-test. ns not significant

USP5 has been shown to play an oncogenic role in many types of cancer,^34–39^ but its role and the underlying mechanism in ESCC are unknown. We thus performed immunofluorescence staining of the ESCC tissue arrays, which revealed a significant positive correlation between USP5 fluorescence intensity and CD31 density (Supplementary Fig. 5a and b). Moreover, patients with high USP5 levels in ESSC had shorter overall survival time (log-rank *P* = 0.005, HR = 1.5; 95% confidence interval = 1.1−2.0; Supplementary Fig. 5c). Next, we carried out gain or loss of *USP5* function in ESCC cell lines (Supplementary Fig. 5d) to examine their effects on angiogenic capacity. As expected, the conditioned medium from *USP5*-knockout ESCC cells markedly suppressed HUVEC migration and tube formation in vitro, whereas that from *USP5*-overexpressed cells promoted these pro-angiogenic phenotypes (Supplementary Fig. 5e−h). In the in vivo experiments, the conditioned medium from *USP5*-knockout ESCC cells reduced hemoglobin content and microvascular density in Matrigel plug tissues (Supplementary Fig. 6a). Additionally, *USP5*-knockout ESCC cells suppressed the growth of xenografts in mice and decreased microvascular density (Supplementary Fig. 6b and c). In contrast, *USP5*-overexpressed promoted all these effects compared with their control cells (Supplementary Fig. 6a, d and e). As the effect of NOTCH1, the effect of USP5 on tumor growth was not due to changes in cell proliferation (Supplementary Fig. 6f−h). Furthermore, we overexpressed *USP5* in *NOTCH1*-knockout cells to further verify the role of USP5 in Notch signaling activation-induced pro-angiogenesis. The results clearly showed that the restoration of *USP5* largely rescued the *NOTCH1*-knockout suppressed pro-angiogenic ability of ESCC cells (Fig. 4a−c and Supplementary Fig. 7a). The results from the mouse xenograft model also showed that the restoration of *USP5* in *NOTCH1*-knockout cells significantly enhanced tumor growth rate and tumor angiogenesis compared with the controls (Fig. 4d−f). However, *USP5* knockout in *NICD1*-overexpressed ESCC cells significantly suppressed the pro-angiogenetic ability of the cells (Supplementary Fig. 7b and c). Together, these findings consistently demonstrate that USP5 is necessary in Notch signaling activation-induced pro-angiogenesis, as summarized in Fig. 4g.

### USP5 deubiquitinates STAT3 to increase the production of pro-angiogenic factors

**Fig. 5:**
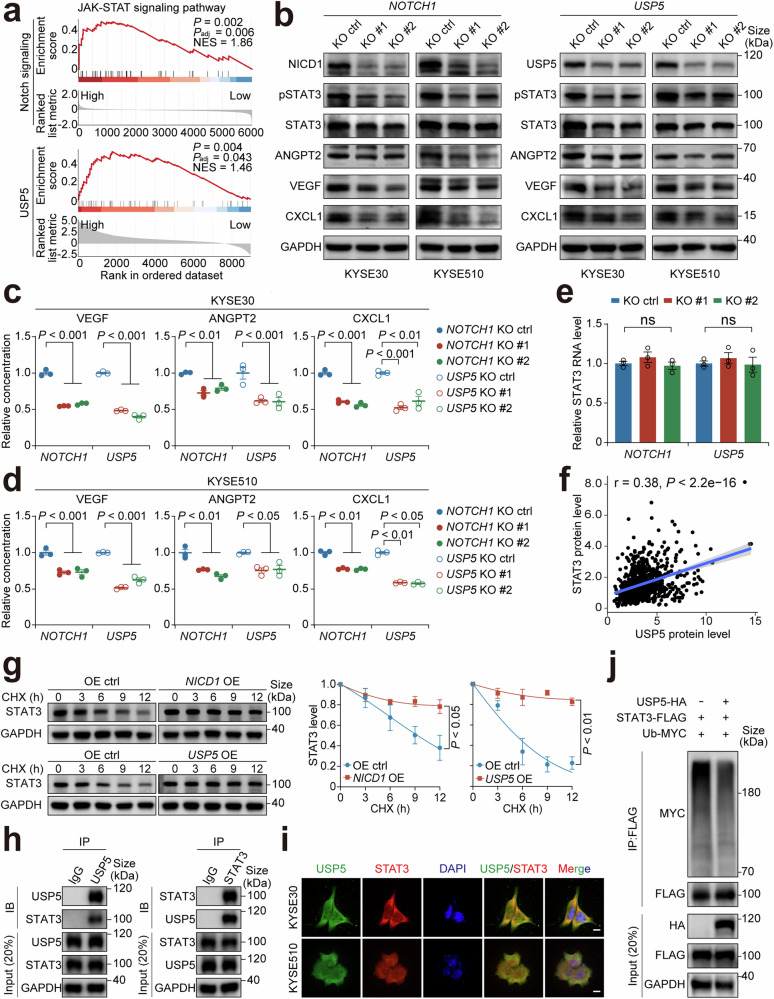
USP5 deubiquitinates STAT3 to increase the production of pro-angiogenic factors. **a** Gene set enrichment analysis revealed significant enrichment of the JAK-STAT signaling pathway in samples with high Notch-signaling score or USP5 level compared with those with low Notch-signaling score or USP5 level. The upper panel shows results from the analysis of our previously published single-cell RNA sequencing data of 60 ESCC samples^28^ based on the Notch-signaling score, and the lower panel shows results from the analysis of a published proteomics data^44^ based on the USP5 level. **b** Western blot analysis shows substantially reduced STAT3, pSTAT3, ANGPT2, VEGF and CXCL1 in *NOTCH1*- or *USP5*-knocked out ESCC cells compared with control cells. The experiment had 3 biological replicates. **c**, **d** Enzyme-linked immunosorbent assays show substantially reduced VEGF, ANGPT2 and CXCL1 levels in the culture medium of *NOTCH1*- or *USP5*-knocked out cells compared with control cells. Data are mean ± SEM from 3 independent measurements. **e** The effects of *NOTCH1* or *USP5* knockout on *STAT3* transcription in ESCC cells. *STAT3* RNA levels were determined by qRT-PCR and normalized by *GAPDH* RNA levels. Data are mean ± SEM from 3 biological replicates. **f** Spearman correlation analysis of the proteomics data^44^ shows a significant and positive correlation between the USP5 levels and STAT3 levels. Shade represents a 95% confidence interval. **g** The effects of *NICD1* or *USP5* overexpression on the STAT3 protein stability in KYSE450 cells in the presence of 50 μg/mL CHX. The left panel shows Western blot analysis of STAT3 protein levels as a function of different CHX treatment time points, and the right panel shows the quantitative statistics. Data are mean ± SEM from three independent measurements. **h** Immunoblot (IB) analysis of the reciprocal coimmunoprecipitation (IP) products with USP5 or STAT3 antibody revealed the interaction of USP5 with STAT3 in KYSE30 cells. **i** Multiplexed immunofluorescence staining and confocal analysis show co-localization of USP5 and STAT3 in ESCC cells. The nuclei were stained with DAPI. Scale bar, 10 μm. **j** STAT3 is deubiquitinated by USP5. Shown is Western blot analysis of cellular lysates from Flag-labeled *STAT3* and MYC-labeled ubiquitin transfected KYSE30 cells with or without HA-labeled *USP5*, followed by treatment with 20 μM MG132 for 6 h. Input was used as control, and the experiment had 3 biological replicates. *P* values in this figure were derived from Student’s *t*-test. ns not significant

Gene set enrichment analysis showed that the JAK-STAT signaling pathway was significantly enriched in Notch signaling score high and USP5 high ESCCs (Fig. 5a). Since STAT3 within the JAK-STAT signaling pathway is known as a key mediator of angiogenesis,^40^ we thus examined the effect of *NOTCH1*-knockout or *USP5*-knockout on the protein levels of STAT3 and its downstream pro-angiogenic molecules in ESCC cells. The results showed that both *NOTCH1* and *USP5* knockout significantly lowed the levels of both total STAT3 and phosphorylated STAT3 (pSTAT3) as well as VEGF, ANGPT2 and CXCL1, which are known to play important roles in angiogenesis^41–43^ (Fig. 5b). In the 4-NQO-induced primary ESCC mouse model, STAT3 expression was also reduced in tumors from *Notch1*^−/−^ mice compared with those from *Notch1*^+/+^ mice (Supplementary Fig. 8a). Furthermore, we also measured the levels of VEGF, ANGPT2 and CXCL1 in the culture medium of ESCC cells using enzyme-linked immunosorbent assays and the results were consistent with that detected by immunoblotting assays (Fig. 5c and d). Multiplexed immunofluorescence staining of the xenografts also showed the consistent changes (Supplementary Fig. 8b). Conversely, *NICD1* and *USP5* overexpression in ESCC cells significantly elevated the levels of these interest molecules (Supplementary Fig. 8c and d). These findings suggest that Notch signaling activation enhances angiogenesis via STAT3 and its downstream pro-angiogenic molecules.

Given the observed activation of STAT3, we first examined whether *NOTCH1* or *USP5* knockout may affect the activity of JAK kinases. Western blotting analysis found that the level of phosphorylated JAK1 or JAK2 was not significantly changed in cells with *NOTCH1* or *USP5* knockout (Supplementary Fig. 9a), suggesting that Notch signaling does not directly affect the phosphorylation of STAT3. Accordingly, we finally focused on examining whether Notch signaling may directly affect STAT3. Previous studies have shown that USP5 is a deubiquitinase and can directly interact with protein molecules such as NLRP3,^34^ SLUG^36^ and PD-1,^39^ inhibiting their ubiquitination and thus enhancing the stability of these proteins. Our results showed that the knockout of *NOTCH1* and *USP5* substantially decreased STAT3 protein but not mRNA levels and the overexpression of *NICD1* and *USP5* substantially increased STAT3 protein but not mRNA levels (Fig. 5b, e, Supplementary Figs. 8c and 9b), suggesting that STAT3 expression may be regulated by USP5 at its post-translation. We also analyzed the published ESCC proteomics data in literature^44^ and found that the USP5 levels were significantly correlated with the STAT3 levels (Fig. 5f). This finding was further validated by immunofluorescence staining of our ESCC tissue array (Supplementary Fig. 9c and d). We then performed biochemical assays to check STAT3 stability under the conditions of *NICD1* or *USP5* overexpression in ESCC cells. The results showed that under cycloheximide (CHX) treatment conditions, either *NICD1* or *USP5* overexpression significantly prolonged STAT3 half-life time, suggesting its increased stability (Fig. 5g). We also performed the immunofluorescence staining and reciprocal coimmunoprecipitation analyses and the results demonstrated co-localization and interaction between USP5 and STAT3 proteins in KYSE30 and KYSE510 cells (Fig. 5h and i). Furthermore, *USP5* overexpression led to a marked reduction in STAT3 polyubiquitination by specifically removing K48-linked, but not K63-linked, polyubiquitin chains (Fig. 5j and Supplementary Fig. 9e). We next ectopically overexpressed *STAT3* in *USP5*-knockout cells to see whether it can rescue the angiogenesis effect. The results from HUVEC migration, tube formation, Matrigel plug, and mouse xenograft experiments showed that ectopic *STAT3* restoration significantly rescued the pro-angiogenesis capacity of *USP5*-knockout ESCC cells (Supplementary Fig. 10a−e). Together, these results strongly suggest that the activation of Notch signaling enhances ESCC tumor angiogenesis through the NOTCH1–USP5–STAT3 axis.

### USP5 is a promising therapy target for ESCC

**Fig. 6:**
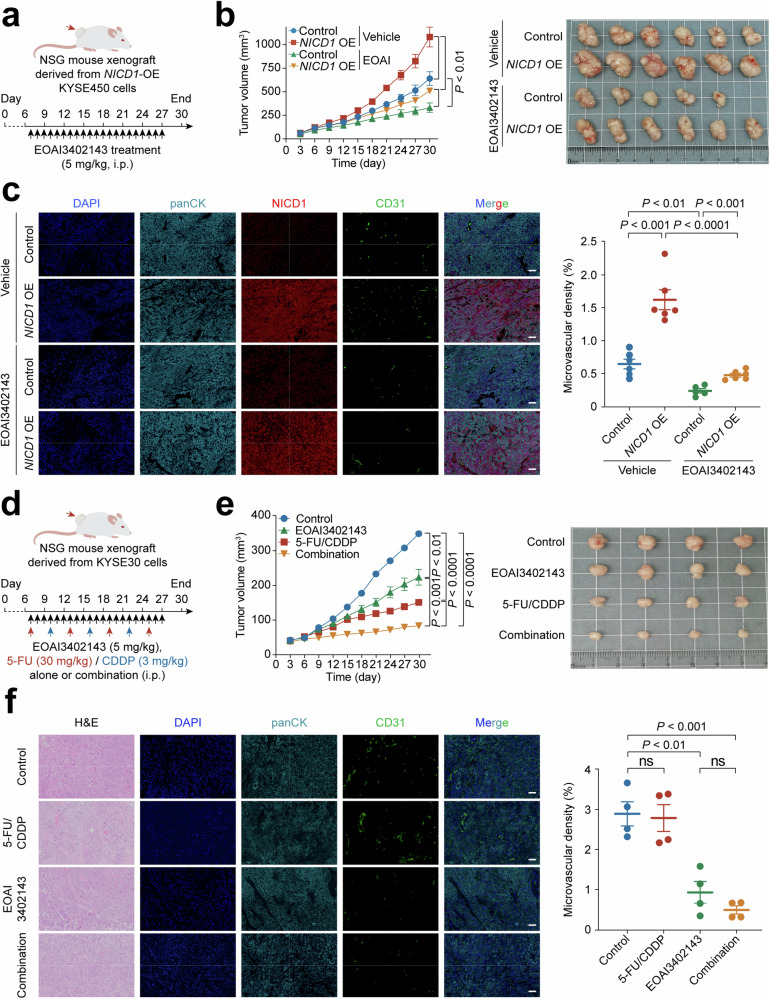
USP5 is a promising therapy target for ESCC. **a** Schematics of USP5 inhibitor EOAI3402143 therapy in NSG mouse ESCC xenograft models. Mice were subcutaneously implanted with *NICD1*-overexpressed KYSE450 cells or their control counterparts and, 7 days later, treated (i.p., daily) with EOAI3402143 or vehicle for 21 days. **b** *NICD1* overexpression significantly promoted, but EOAI3402143 treatment significantly suppressed the growth rates of mouse ESCC xenografts. The left panel shows growth curves of xenografts with different treatments, and the right panel shows tumors obtained from mice at the end of the experiment (day 30). Data are mean ± SEM from 5 or 6 mice. **c** Multiplexed immunofluorescence staining analysis of NICD1 and CD31 levels in xenografts with different treatments. The left panel shows immunofluorescence staining images, and the right panel shows quantitative statistics. The ESCC xenograft tumor was identified by panCK staining. Scale bar, 50 μm. Data are mean ± SEM from 5 or 6 mice. **d** Schematics of USP5 inhibitor EOAI3402143 combined with 5-FU/CDDP therapy in NSG mouse ESCC xenograft models. Mice were subcutaneously implanted with KYSE30 cells, and 7 days later, treated with EOAI3402143 or 5-FU/CDDP, or their combination. **e** EOAI3402143 treatment, especially in combination with 5-FU/CDDP, significantly suppressed the growth of mouse ESCC xenografts. The left panel shows growth curves of xenografts with different treatments, and the right panel shows tumors obtained from mice at the end of the experiment (day 30). Data are mean ± SEM from 4 mice. **f** Multiplexed immunofluorescence staining analysis of CD31 levels in xenografts with different treatments. The left panel shows H&E and immunofluorescence staining images, and the right panel shows quantitative statistics. The ESCC xenograft tumor was identified by panCK staining. Scale bar, 50 μm. Data are mean ± SEM from 4 mice. *P* values in this figure were derived from Student’s *t*-test. ns not significant

Since angiogenesis is critical for tumor development and metastasis, and our findings identify the NOTCH1–USP5–STAT3 axis as a key pro-angiogenic pathway in ESCC, we sought to evaluate its therapeutic potential. Although NOTCH inhibitors, such as γ-secretase inhibitors and monoclonal antibodies, have been tested in both preclinical and clinical settings, their application has been limited by off-target effects, low selectivity, and considerable toxicity.^19^ Given that USP5 functions as a critical downstream effector of NOTCH1 and contributes to the upregulation of multiple pro-angiogenic factors, we supposed that targeting USP5 might offer a more selective strategy to disrupt this pathway while avoiding the broad systemic inhibition of Notch signaling. We therefore assessed the anti-tumor efficacy of the USP5 inhibitor, EOAI3402143, an inhibitor has shown to possess the anti-tumor activity in several preclinical models, including colorectal cancer, melanoma, and B-cell malignancies.^39,45,46^ We first established mouse subcutaneous xenograft models using *NICD1*-overexpressed human ESCC cells and treated the mice with EOAI3402143, as shown in Fig. 6a. The results showed that xenografts derived from *NICD1*-overexpresed KYSE450 cells had significantly larger sizes than xenografts derived from control cells. EOAI3402143 treatment had significant inhibitory effect on the growth of both *NICD1*-overexpresed and *NICD1*-nonoverexpressed tumors, with the efficacy of the inhibitor being substantially larger to *NICD1*-overexpressed tumors than to *NICD1*-nonoverexpressed tumors (Fig. 6b). Compared with mice in the vehicle group, mice treated with EOAI3402143 did not show significant difference in body weight gain (Supplementary Fig. 11a). Immunofluorescence analysis of the xenograft tissues revealed that the tumor microvascular density was much higher in *NICD1*-overexpressed xenografts than in *NICD1*-non-overexpressed ones and this high angiogenesis in tumors could be completely suppressed by EOAI3402143 treatment (Fig. 6c). Consistently, EOAI3402143 also reduced STAT3 expression in xenografts (Supplementary Fig. 11b). In addition to USP5, EOAI3402143 also inhibits other deubiquitinases, including USP9X and USP24,^45^ so we used siRNA to individually knock down each enzyme in ESCC cells. Neither *USP9X* nor *USP24* knockdown altered total or phosphorylated STAT3 levels, and reciprocal coimmunoprecipitation analysis showed no direct interaction between STAT3 and either enzyme (Supplementary Fig. 11c and d). These results indicate that EOAI3402143 suppresses tumor angiogenesis by inhibiting the USP5–STAT3 axis.

As tumor angiogenesis plays an important role in chemotherapy resistance and anti-angiogenic agents were usually combined with chemotherapeutics in the clinic,^47^ we thus assessed whether inhibiting USP5 may enhance the antitumor efficacy of chemotherapy in ESCC (Fig. 6d). As a result, we found that compared with vehicle control and single drug treatment, combined EOAI3402143 and 5-fluorouracil (5-FU)/cisplatin (CDDP) treatment greatly inhibited both tumor growth rate (Fig. 6e) and tumor angiogenesis (Fig. 6f), without significantly affecting mouse body weight gain (Supplementary Fig. 11e). STAT3 expression in xenografts was also markedly reduced in the EOAI3402143-only and combination treatment groups (Supplementary Fig. 11f). These results indicated that Notch signaling inhibitors enhanced the anticancer effect of chemotherapeutic agents in treating ESCC with activated Notch signaling, suggesting that the NOTCH1–USP5–STAT3 axis may be a promising therapeutic target.

## Discussion

Notch signaling plays complex and context-dependent roles in cancer, with its function in ESCC being particularly debated. The multifaceted roles of Notch signaling in various types of cancer have been documented. For instance, in non-small cell lung cancer, NOTCH1 activates a positive feedback loop with its downstream target RFC4, promoting metastasis and stemness traits in tumor cells.^20^ In hepatocellular carcinoma, NOTCH1 drives EMT and metastasis through the RNF187 activation.^21^ However, the role of Notch signaling in ESCC remains controversial, with studies reporting divergent effects depending on the stage and context of the cancer. Some studies have suggested that *NOTCH1* mutations drive clonal expansion in normal aged esophageal epithelium but impair tumor growth^17^; the other has reported that the interplay between NOTCH1 and NOTCH3 promotes EMT and tumor initiation, supporting a pro-tumorigenic role for Notch signaling in tumor initiation phase.^22^ However, a previous study has also shown that JAG1/2-mediated Notch signaling maintains esophageal homeostasis and suppresses foregut tumorigenesis by restricting the basal progenitor cell pool, suggesting a potential tumor-suppressive role of Notch signaling in early tumorigenesis.^48^ These controversial findings warrant further investigations.

In the present study, we have specifically focused on the role of Notch signaling in the late stages of ESCC, where we have revealed that it may promote tumor progression. We have demonstrated that the activated Notch signaling enhances angiogenesis in ESCC by inducing endothelial cell migration and tube formation that are essential for new blood vessel formation. Angiogenesis is a crucial process for tumor growth and metastasis, as it ensures an adequate supply of oxygen and essential nutrients and facilitates the entry of tumor cells into the bloodstream. Our results in the present study highlight the pivotal role of Notch signaling in regulating ESCC angiogenesis. Since most *NOTCH1* mutations in ESCC occur in the EGF-like domain, which impedes its ligand from binding to it, leading to Notch signaling inactivation,^15^ one may expect that *NOTCH1*-mutated cells will remain in aged normal esophagus but be largely eliminated in ESCC tumors due to the angiogenetic need of cancer. This perception is consistent with our previous study on genomics and clone evolution of multi-stage ESCC development, showing that *NOTCH1* mutant clones are prevalent in normal and precancerous esophageal epithelium but vanish mostly in ESCC.^8^ More importantly, in the present study, we have further demonstrated that targeting the NOTCH1 signaling pathway, such as the USP5 enzyme, holds significant therapeutic potential for ESCC, in line with the important role of this angiogenesis pathway in ESCC.

**Fig. 7:**
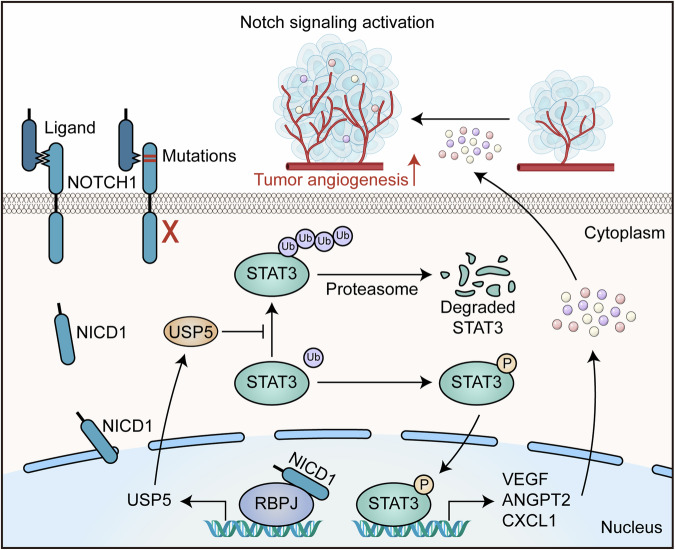
Schematics of the proposed role of Notch-signaling activation in enhancing tumor angiogenesis in ESCC. The aberrantly high Notch signaling transcriptionally activates the *USP5* gene to produce USP5, which deubiquitinates STAT3 to prevent STAT3 from ubiquitination and degradation. The increased STAT3 activity enhances the production of its downstream pro-angiogenic factors such as VEGF, ANGPT2, and CXCL1, resulting in angiogenesis in ESCC. Thus, the NOTCH1–USP5–STAT3 axis might be a therapeutic target for ESCC

We have revealed that ubiquitin-specific protease USP5 is an effector molecule downstream of the Notch signaling that induces ESCC angiogenesis. Ubiquitination modifies target proteins by attaching ubiquitin, leading to their proteasome-mediated degradation. Ubiquitination has been shown to play critical roles in various biological processes, including DNA damage repair, cell cycle regulation, and apoptosis.^49^ The dynamic balance between ubiquitination and deubiquitination is tightly regulated by E3 ubiquitin ligases and deubiquitinases. As an important member of the deubiquitinase family, USP5 has been suggested to be an oncoprotein in several types of cancer, involved in regulating the stability of proteins in some oncogenic signaling pathways.^34–39^ For example, USP5 may stabilize the ERK signaling, which governs the PD-1 homeostasis via deubiquitination, and thus influences cancer immunotherapy outcomes.^39^ The USP5–Beclin1 axis has been reported to override p53-dependent senescence, promoting Kras-induced lung tumorigenicity in mice.^35^ However, the role of USP5 in tumor angiogenesis is rarely known. In the present study on ESCC, we have demonstrated for the first time that USP5 is an effector enzyme in Notch signaling activation-induced tumor angiogenesis. The NOTCH1 signaling activation-produced NICD1 transcriptionally upregulates *USP5*, which consequently prevents ubiquitination degradation of STAT3 by specifically removing K48-linked polyubiquitin chains of STAT3. The increased STAT3 enhances the expression and secretion of pro-angiogenic factors, including VEGF, ANGPT2, and CXCL1, which promote ESCC angiogenesis. Conversely, in cells with loss-of-function *NOTCH1* mutations, Notch signaling cannot be activated and thus fails to promote angiogenesis via this mechanism (Fig. 7). These findings underscore a novel function of USP5 in mediating tumor angiogenesis induced by Notch signaling.

The results in the current study not only deepen our understanding of the molecular mechanisms driving angiogenesis in ESCC but also suggest USP5 as a promising therapeutic target for disrupting tumor angiogenesis. An imbalance between pro-angiogenic and anti-angiogenic factors within the TME can lead to abnormal tumor vasculature, accelerating tumor growth. Anti-angiogenic therapy combined with chemotherapy has already been included in treatment guidelines for advanced ESCCs; however, patient benefit remains limited.^50^ This is likely due to the complexity of tumor vasculature and the challenges in achieving sustained therapeutic effects. Due to the off-target effects, low selectivity, and considerable toxicity of NOTCH inhibitors, we chose to target USP5 in the present study to evaluate its antitumor efficacy. We have found that the use of the USP5 inhibitor EOAI3402143, especially when combined with chemotherapy agents 5-FU and CDDP, can significantly suppress angiogenesis and tumor growth, and this therapeutic strategy has superior efficacy compared with 5-FU/CDDP alone. These results suggest that targeting USP5 in combination with chemotherapy may offer a promising new strategy to improve ESCC treatment outcomes.

Although we have achieved several advances as summarized above, our study has some limitations. We have provided strong evidence for the pro-angiogenic role of Notch signaling in ESCC tumors, but the precise function of this signaling throughout ESCC tumorigenesis, particularly its effects on the early versus late stages of tumorigenesis, needs further elucidation. More studies are also warranted to fully elucidate the potential dual roles of the Notch signaling pathway in both tumor initiation and progression. Additionally, while our findings highlight USP5 as a critical downstream target of Notch signaling in ESCC, its broader role in the disease beyond promoting angiogenesis is yet to be fully understood. Thus, it would be interesting to examine the role of USP5 in other cancer malignant phenotypes, such as metastasis and immune evasion, to better assess its therapeutic potential.

In summary, the present study has revealed that Notch signaling activation enhances tumor angiogenesis in ESCC through the NOTCH1–USP5–STAT3 axis. Targeting USP5 might thus offer a new avenue for anti-angiogenic therapies to improve the treatment of ESCC and other cancers where angiogenesis plays a critical role, which is warranted for clinical validation.

## Materials and methods

### Single-cell RNA sequencing data analysis

We reanalyzed our previously published ESCC single-cell RNA sequencing data available in the GEO database (GSE160269) for the purpose of the present study. Quality control and downstream analysis were performed using the Seurat package (v4.3.0), with batch effects corrected using the Harmony package (v0.1.1). Cell types were annotated based on well-established marker genes: EPCAM, SFN, KRT4, KRT5, KRT19, and KRT14 for epithelial cells; FN1, DCN, COL1A1, COL1A2, COL3A1, and COL6A1 for fibroblasts; and VWF, PECAM1, ENG, PLVAP, and RAMP2 for endothelial cells. Notch signaling and angiogenesis signature scores for individual cells were calculated by integrating hallmark genes associated with each pathway using Seurat’s AddModuleScore function. Gene set enrichment analysis and gene ontology (GO) pathway enrichment analysis were performed using the R package clusterProfiler (v4.6.2).

### Human ESCC tumor samples collection

For clinical validation, we collected ESCC tumor samples from patients who underwent surgery at Linzhou Cancer Hospital, Henan Province, China, between 2016 and 2019. The study was approved by the ethics committee of the Cancer Hospital, Chinese Academy of Medical Sciences (NCC23/305-4047), and all patients provided written informed consent. Fresh tumor tissues were formalin-fixed and paraffin-embedded. Histopathological diagnosis was performed based on hematoxylin and eosin (H&E) staining of tissue sections and independently confirmed by at least two pathologists. Representative tumor regions were selected to construct tissue microarrays. Clinical data were obtained from electronic medical records, and survival information was collected via standardized telephone follow-up. All specimens and associated data were anonymized.

### Immunohistochemistry and multiplexed immunofluorescence staining analysis

Formalin-fixed paraffin-embedded tissue sections were first deparaffinized twice in Histo-Clear II and then rehydrated through a graded ethanol series. Endogenous peroxidase activity was quenched, and antigen retrieval was performed. Sections were incubated overnight at 4 °C with primary antibodies against cleaved Notch1 (Cell Signaling Technology), CD31 (Proteintech), USP5 (Abcam), STAT3 (Cell Signaling Technology), Ki-67 (Abcam), VEGF (Proteintech), Angiopoietin 2 (Proteintech) and CXCL1 (Proteintech).

For immunohistochemistry staining, sections were subsequently incubated with horseradish peroxidase (HRP)-conjugated secondary antibodies at room temperature for 20 minutes. The signal was developed using 3,3’-diaminobenzidine (DAB) substrate and counterstained with hematoxylin.

For multiplexed immunofluorescence staining, the Opal 5-Color Manual IHC Kit (PANOVUE) was used. Following primary antibody incubation, secondary antibodies and tyramide signal amplification (TSA)-conjugated fluorophores (Opal 480, Opal 520, Opal 570, and Opal 650) were applied sequentially. The slides were counterstained with DAPI to visualize the nuclei and mounted with mounting medium.

Imaging was performed using a NanoZoomer slide scanner, and image analysis was conducted with ImageJ software. For quantitative assessment, the average staining intensity was calculated from three randomly selected, non-overlapping regions per sample. All procedures, including staining, imaging, and quantification, were performed in a blinded manner to minimize bias.

### Analysis of the correlation of NICD1 with patient survival time and clinical characteristics

The Kaplan-Meier method and log-rank test were used to perform the overall survival time analysis on 312 ESCC patients categorized into high and low NICD1 level based on the median NICD1 immunofluorescence intensity. The survfit function from the survival package was used to obtain the *P* value for the log-rank test. The coxph function in the survival package was used to calculate the hazard ratios and their 95% confidence intervals using age, gender, smoking, drinking, and pathological TNM stage as the covariates. The correlation between NICD1 levels and patient clinical indicators was assessed using the Chi-square test.

### Cell lines and cell culture

Human umbilical vein endothelial cells (HUVECs) were obtained from AOYINBIO (Shanghai, China) and cultured in Endothelial Cell Medium (ScienCell) supplemented with endothelial cell growth supplement (ECGS). KYSE30, KYSE510, KYSE450, and KYSE150 ESCC cell lines, generously provided by Dr. Y. Shimada from the Hyogo College of Medicine in Japan, were cultured in DMEM (Corning). DNA fingerprinting was used to authenticate all cell lines, and they were also screened to ensure free of mycoplasma contamination. All the cells were incubated at 37°C in a humid atmosphere with 5% CO_2_.

### Establishment of the interest gene knockout or overexpression cell lines

We generated stable *NOTCH1*- or *USP5*-knockout cell lines (KYSE30 and KYSE510) using specific single-guide RNAs (sgRNAs) targeting *NOTCH1* or *USP5* designed by Genechem. The *NOTCH1*-specific sgRNAs were cloned into the GV708 vector (U6-sgRNA-EF1a-Cas9-FLAG-CMV-EGFP-P2A-puro) while *USP5*-specific sgRNAs were cloned into the GV823 vector (U6-sgRNA-EF1a-Cas9-FLAG-P2A-Blasticidin). We also established *NICD1*, *USP5*, or STAT3-overexpressed cell lines (KYSE30, KYSR510, KYSE450 and KYSE150). *NOTCH1* (NM_017617(1754-2555aa)), *USP5* (NM_001098536.2-HA), or *STAT3* (NM_139276.3-Flag) sequences were cloned into the GV492 (Ubi-MCS-3FLAG-CBh-gcGFP-IRES-puromycin), GV341 (Ubi-MCS-3FLAG-SV40-puromycin), or GV301 (Ubi-MCS-SV40-blasticidin) vectors, respectively. The used sequences are listed in Supplementary Table 3 and the lentivirus was packed by Genechem.

### Western blot analysis

Total protein was extracted from cells using RIPA lysis buffer (Solarbio) supplemented with Protease and Phosphatase Inhibitor Cocktail (NCM Biotech). Protein concentration was measured using the BCA assay kit (Thermo Fisher Scientific). Protein samples (about 20 μg) were separated by SDS–PAGE and transferred to a PVDF membrane (Millipore). Antibody against cleaved NOTCH1, STAT3, phosphorylated-STAT3 (Tyr705), HIF2α, JAK1, phosphorylated-Jak1 (Tyr1034/1035), JAK2 and phosphorylated-JAK2(Tyr1007/1008) were from Cell Signaling Technology and antibody against HIF1α, CXCL1, VEGFA, Angiopoietin 2, HA tag, Myc tag, His tag, USP9X and USP24 were from Proteintech. Antibodies against USP5 or Flag tag were from Abcam or Sigma-Aldrich. Membranes were incubated overnight at 4 °C with primary antibody, followed by 2 h of incubation with secondary antibody (Proteintech) at room temperature. Protein signals were visualized using the Amersham Imager 600, with detection via chemiluminescence using the Super Sensitive ECL luminescence reagent (MeilunBio).

### Conditioned medium preparation

We prepared a conditioned medium from the cultivation of various gene-operated ESCC cells. Cells cultured in a six-well plate and grown to reach ~70% confluency were washed twice with PBS and incubated again in a serum-free medium for 24 h. The supernatant of culture medium was collected by centrifuging for 10 min at 2000×*g* to yield conditioned medium, which was used immediately or stored at −80 °C for future use.

### HUVEC migration assay

HUVECs were suspended in serum-free medium and seeded into the upper chamber of a 24-well Transwell system with 8-μm pore polycarbonate filters (Corning). The conditioned medium (500 μL) prepared as described above was added to the lower chamber. After incubating for 24 h, cells that migrated to the lower surface of the membrane were fixed with 100% methanol and stained with crystal violet. The stained cells were imaged under a microscope, and the migration area was quantified by measuring the area covered by migrated cells in five randomly selected non-overlapping visual fields.

### HUVEC tube formation assay

A 50-μL volume of precooled growth factor-reduced phenol red-free Matrigel (Corning) was added to each well of a 24-well plate and polymerized at 37 °C for 30 min. HUVECs (5 × 10^4^) suspended in 500 μL of the conditioned medium were seeded into each well and cultured for 3 h. To evaluate capillary network formation, HUVECs were stained with Calcein AM (Beyotime). Images of randomly selected fields were captured, and the branching length and the number of tubes were quantified.

### Matrigel plug assay in mice

A 100-µL volume of the conditioned medium prepared as described above was mixed with 350-µL of growth factor-reduced Matrigel (Corning). The mixture was subcutaneously injected into the midline of the back of C57BL/6 mice. One week after the injection, the Matrigel plugs were excised, and the hemoglobin content and microvascular density in the tissue were assessed.

### Animal experiments

Mice used in this study were maintained in a pathogen-free environment at the animal research facility, and the experiment procedures were approved by the Institutional Animal Care and Use Committee of the Chinese Academy of Medical Sciences (NCC2021A271).

For the subcutaneous xenograft mouse model, KYSE30 cells (3 × 10^6^) or KYSE450 (4 × 10^6^) cells, suspended in 100-μL of PBS, were subcutaneously injected into the hind legs of 6-week-old male NOD/SCID/IL-2Rγnull (NSG) mice. Tumor growth was monitored by palpation, and tumor size and body weight were measured every 3 days. Tumor volumes were calculated using the formula length × width² × 0.5.

For drug treatment, mice were divided into groups as indicated in the corresponding figure legends. For the experiment shown in Fig. 6a, mice bearing xenograft tumors derived from *NICD1*-overexpressed or non-overexpressed KYSE450 cells were treated with EOAI3402142 dissolved in 2% DMSO/40% PEG300/5% Tween/53% PBS (5 mg/kg, i.p., daily) for 21 doses. For the experiment shown in Fig. 6d, mice bearing xenograft tumor derived from KYSE30 cells were respectively treated via i.p. with EOAI3402142 (5 mg/kg, daily), 5-FU (30 mg/kg)/CDDP (3 mg/kg) dissolved in PBS (every 6 days), or combination of these two regimens. Mice were sacrificed, and tumors were removed at the time endpoint as indicated in the figure legends. The same volume of vehicle was used as the control.

For the 4-NQO-induced primary ESCC mouse model, the experimental procedure was conducted as previously described.^29^ *Notch1* homozygous knockout (*Notch1*^−/−^: *ED-L2*-*Cre*; *Notch1*^L/L^) and wild-type mice were used in the study, with genotypes confirmed via PCR. Mice were administered 4-NQO (Sigma-Aldrich) in drinking water at a concentration of 100 μg/mL for 16 weeks. The carcinogen-containing water was freshly prepared and replenished weekly, and the mice had free access to the treated water throughout the treatment period. After the 16-week exposure, 4-NQO was withdrawn and replaced with sterile water for over 10 weeks.

### In vitro cell proliferation assay (CCK-8)

KYSE30 and KYSE510 cells were seeded into 96-well plates at a density of 2 × 10^3^ cells per well. Cell proliferation was assessed at 0, 24, 48, 72, 96, and 120 h using the Cell Counting Kit-8 (CCK-8, DOJINDO). At each time point, 10-μL of CCK-8 reagent was diluted 1:10 in serum-free medium to a final volume of 100-μL and added to each well, followed by incubation for 1 h at 37 °C. The absorbance at 450 nm was measured using a microplate reader. A proliferation curve was generated based on the optical density values.

### CUT&Tag sequencing and data analysis

We performed the CUT&Tag sequencing using the CUT&Tag Assay Kit (Vazyme). Tumor cell nuclei were isolated and incubated with concanavalin A-coated magnetic beads for 10 min, followed by overnight incubation at 4 °C with either an anti-cleaved NOTCH1 antibody (Cell Signaling Technology) or control IgG. On the following day, the beads were washed and incubated with a secondary antibody for 1 h. Hyperactive pA/G Transposase was then added and incubated for 1 h to facilitate tagmentation. The tagmented DNA was resuspended in tagmentation buffer and purified using Proteinase K digestion and DNA Extract Beads. The resulting DNA was amplified to generate sequencing libraries, which were subjected to paired-end 150-bp sequencing on the Illumina NovaSeq 6000 platform (Annoroad Gene Company). Raw sequencing data were processed using standard bioinformatics tools. Sequence alignment was performed with Bowtie2 (v2.5.1), aligning to the GRCh38 reference genome (GENCODE v41). Peak calling was carried out using MACS3 (v3.0.0) to identify enriched chromatin-binding regions. Motif analysis was conducted using HOMER (v4.11) to identify enriched transcription factor binding motifs within NICD1 CUT&Tag peak regions. Further analysis included peak visualization and genomic annotation using IGV (v2.16.2).

### RNA sequencing and data analysis

Total RNA was extracted from cells using TRIzol reagent (Invitrogen). The library was prepared with the VAHTS Universal V6 RNA-Seq Library Prepkit. RNA sequencing was conducted on the Illumina NovaSeq 6000 platform. The raw sequencing data were processed by adapter trimming and quality control using Trim Galore (v0.6.6). Cleaned and filtered reads were then quantified using the pseudo-alignment tool Salmon (v1.2.0) and aligned to the GRCh38 reference genome. Differentially expressed genes were identified using DESeq2 (v1.38.3).

### Quantitative real-time PCR analysis

Total RNA was extracted using the RNA-Quick Purification Kit (ES Science) and subsequently reverse-transcribed with the PrimeScript RT Reagent Kit (Takara). The mRNA levels were detected in Quantagene q900 qPCR system (Kubo Technology) with TB Green® Premix Ex TaqTM II (Takara) and the corresponding primers (Supplementary Table 3). The target gene expression was calculated using the ΔΔCt method relative to *GAPDH* mRNA.

### Chromatin immunoprecipitation-coupled quantitative PCR analysis

The assays were performed using the SimpleChIP® Plus Sonication Chromatin IP Kit (Cell Signaling Technology). KYSE30 and KYSE510 cells were initially treated with 1% formaldehyde for 10 min to crosslink proteins to DNA, followed by a 5-min incubation with glycine to quench the reaction. The cells were then collected, lysed with cell lysis and nuclear lysis buffers, and the chromatin was sheared using the Covaris S220 Focused-ultrasonicator for 600 s at 4 °C). After shearing, the chromatin was incubated with antibody against cleaved NOTCH1, RBPSUH, Histone H3, or IgG. Finally, the DNA was purified and analyzed by qPCR with the primers for the *USP5* promoter (Supplementary Table 3).

### Reporter gene assay

The *USP5* promoter region, ~3000 bp upstream of the transcription start site, was amplified by PCR and cloned into the GV238 vector (MCS-firefly Luciferase). The plasmid construct containing the wild-type (WT) *USP5* promoter with the RBPJ binding motif or the mutant-type (MUT) *USP5* promoter with deletion of the RBPJ binding motif was designed by Genechem. The plasmids were then transfected into KYSE30 and KYSE510 cells using jetPRIME® (Polyplus). When the cells' growth reached 80–90% confluency, luciferase reporter assays were performed according to the instructions (Promega).

### Enzyme-linked immunosorbent assay

The concentrations of VEGF, CXCL1, and Angiopoietin 2 in cell culture medium were measured using enzyme-linked immunosorbent assay kits from Proteintech according to the manufacturer’s instructions.

### Immunoprecipitation assay

Total proteins were extracted from cells using a weak RIPA lysis buffer (Beyotime) supplemented with a Protease and Phosphatase Inhibitor Cocktail (NCM Biotech). We performed USP5 and STAT3 immunoprecipitation using Dynabeads™ Protein G (Invitrogen), and the eluted product was analyzed by Western blot.

### Statistical analysis

R version 4.1.2 and GraphPad Prism 9 were used to perform the statistical analysis. Statistical details and methods are provided in the figure legends, main text, or methods section. The *P* values were determined using either the Wilcoxon rank-sum test or Student’s *t*-test. Specifically, the Wilcoxon rank-sum test was employed to assess whether there was a significant difference between two non-normally distributed quantitative datasets. For functional assays, the comparison between two treatment groups was made using Student’s *t*-test. The difference in patient survival times was estimated with Kaplan–Meier method, and the *P* value was derived from the log-rank test. The hazard ratio was calculated using multivariate Cox proportional hazard models.

## Supplementary information

Supplementary files

## Data Availability

The two published datasets are available in the GEO Database (GSE160269 and GSE199654). The raw data from the CUT&Tag and RNA sequencing of ESCC cell lines have been deposited in the NCBI Sequence Read Archive under accession numbers PRJNA1272810 and PRJNA1273102. All other data and information supporting the findings are available in the article and the Supplementary files. The original code is accessible from the corresponding author upon reasonable request.

## References

[1] Siegel, R. L., Giaquinto, A. N. & Jemal, A. Cancer statistics, 2024. *CA Cancer J. Clin.* **74**, 12–49 (2024). 38230766 10.3322/caac.21820

[2] Abnet, C. C., Arnold, M. & Wei, W. Epidemiology of esophageal squamous cell carcinoma. *Gastroenterology* **154**, 360–373 (2018). 28823862 10.1053/j.gastro.2017.08.023 PMC5836473

[3] Smyth, E. C. et al. Oesophageal cancer. *Nat. Rev. Dis. Prim.* **3**, 1–21 (2017). 10.1038/nrdp.2017.48 PMC6168059 28748917

[4] Yang, X. et al. Collagen 1-mediated CXCL1 secretion in tumor cells activates fibroblasts to promote radioresistance of esophageal cancer. *Cell Rep.* **42**, 113270 (2023). 37851572 10.1016/j.celrep.2023.113270

[5] Chen, X. et al. Alarmin S100A8 imparts chemoresistance of esophageal cancer by reprogramming cancer-associated fibroblasts. *Cell Rep. Med.* **5**, 101576 (2024). 38776909 10.1016/j.xcrm.2024.101576 PMC11228400

[6] Puhr, H. C., Prager, G. W. & Ilhan-Mutlu, A. How we treat esophageal squamous cell carcinoma. *ESMO Open* **8**, 100789 (2023). 36791637 10.1016/j.esmoop.2023.100789 PMC9958251

[7] Chang, J. et al. Genomic analysis of oesophageal squamous-cell carcinoma identifies alcohol drinking-related mutation signature and genomic alterations. *Nat. Commun.* **8**, 15290 (2017). 28548104 10.1038/ncomms15290 PMC5477513

[8] Chang, J. et al. Genomic alterations driving precancerous to cancerous lesions in esophageal cancer development. *Cancer Cell* **41**, 2038–2050.e5 (2023). 38039962 10.1016/j.ccell.2023.11.003

[9] Xi, Y. et al. Multi-omic characterization of genome-wide abnormal DNA methylation reveals diagnostic and prognostic markers for esophageal squamous-cell carcinoma. *Signal Transduct. Target. Ther.* **7**, 1–13 (2022). 35210398 10.1038/s41392-022-00873-8 PMC8873499

[10] Song, Y. et al. Identification of genomic alterations in oesophageal squamous cell cancer. *Nature* **509**, 91–95 (2014). 24670651 10.1038/nature13176

[11] Cui, Y. et al. Whole-genome sequencing of 508 patients identifies key molecular features associated with poor prognosis in esophageal squamous cell carcinoma. *Cell Res.* **30**, 902–913 (2020). 32398863 10.1038/s41422-020-0333-6 PMC7608103

[12] Gao, Y. et al. Genetic landscape of esophageal squamous cell carcinoma. *Nat. Genet.* **46**, 1097–1102 (2014). 25151357 10.1038/ng.3076

[13] Lin, D. et al. Genomic and molecular characterization of esophageal squamous cell carcinoma. *Nat. Genet.* **46**, 467–473 (2014). 24686850 10.1038/ng.2935 PMC4070589

[14] Li, R. et al. A body map of somatic mutagenesis in morphologically normal human tissues. *Nature* **597**, 398–403 (2021). 34433965 10.1038/s41586-021-03836-1

[15] Martincorena, I. et al. Somatic mutant clones colonize the human esophagus with age. *Science* **362**, 911–917 (2018). 30337457 10.1126/science.aau3879 PMC6298579

[16] Colom, B. et al. Spatial competition shapes the dynamic mutational landscape of normal esophageal epithelium. *Nat. Genet.* **52**, 604–614 (2020). 32424351 10.1038/s41588-020-0624-3 PMC7116672

[17] Abby, E. et al. Notch1 mutations drive clonal expansion in normal esophageal epithelium but impair tumor growth. *Nat. Genet.* **55**, 232–245 (2023). 36658434 10.1038/s41588-022-01280-z PMC9925379

[18] Majumder, S. et al. Targeting Notch in oncology: the path forward. *Nat. Rev. Drug Discov.* **20**, 125–144 (2021). 33293690 10.1038/s41573-020-00091-3

[19] Zhou, B. et al. Notch signaling pathway: architecture, disease, and therapeutics. *Signal Transduct. Target. Ther.* **7**, 1–33 (2022). 35332121 10.1038/s41392-022-00934-y PMC8948217

[20] Liu, L. et al. An RFC4/Notch1 signaling feedback loop promotes NSCLC metastasis and stemness. *Nat. Commun.* **12**, 2693 (2021). 33976158 10.1038/s41467-021-22971-x PMC8113560

[21] Zhang, L. et al. An essential role of RNF187 in Notch1 mediated metastasis of hepatocellular carcinoma. *J. Exp. Clin. Cancer Res.* **38**, 384 (2019). 31477177 10.1186/s13046-019-1382-x PMC6720101

[22] Natsuizaka, M. et al. Interplay between Notch1 and Notch3 promotes EMT and tumor initiation in squamous cell carcinoma. *Nat. Commun.* **8**, 1758 (2017). 29170450 10.1038/s41467-017-01500-9 PMC5700926

[23] Hanahan, D. Hallmarks of cancer: new dimensions. *Cancer Discov.* **12**, 31–46 (2022). 35022204 10.1158/2159-8290.CD-21-1059

[24] Leung, D. W., Cachianes, G., Kuang, W.-J., Goeddel, D. V. & Ferrara, N. Vascular endothelial growth factor is a secreted angiogenic mitogen. *Science* **246**, 1306–1309 (1989). 2479986 10.1126/science.2479986

[25] Montesano, R., Vassalli, J. D., Baird, A., Guillemin, R. & Orci, L. Basic fibroblast growth factor induces angiogenesis in vitro. *Proc. Natl Acad. Sci. USA* **83**, 7297–7301 (1986). 2429303 10.1073/pnas.83.19.7297 PMC386703

[26] Stratmann, A., Risau, W. & Plate, K. H. Cell Type-specific expression of angiopoietin-1 and angiopoietin-2 suggests a role in glioblastoma angiogenesis. *Am. J. Pathol.* **153**, 1459–1466 (1998). 9811337 10.1016/S0002-9440(10)65733-1 PMC1853417

[27] Cao, Y., Langer, R. & Ferrara, N. Targeting angiogenesis in oncology, ophthalmology and beyond. *Nat. Rev. Drug Discov.* **22**, 476–495 (2023). 37041221 10.1038/s41573-023-00671-z

[28] Zhang, X. et al. Dissecting esophageal squamous-cell carcinoma ecosystem by single-cell transcriptomic analysis. *Nat. Commun.* **12**, 5291 (2021). 34489433 10.1038/s41467-021-25539-x PMC8421382

[29] Chang, J. et al. Single-cell multi-stage spatial evolutional map of esophageal carcinogenesis. *Cancer Cell* **43**, 380–397.e7 (2025). 40068596 10.1016/j.ccell.2025.02.009

[30] Jiang, N. et al. HIF-1ɑ-regulated miR-1275 maintains stem cell-like phenotypes and promotes the progression of LUAD by simultaneously activating Wnt/β-catenin and Notch signaling. *Theranostics* **10**, 2553–2570 (2020). 32194819 10.7150/thno.41120 PMC7052895

[31] Hu, Y. et al. Hif-1α and Hif-2α differentially regulate Notch signaling through competitive interaction with the intracellular domain of Notch receptors in glioma stem cells. *Cancer Lett.* **349**, 67–76 (2014). 24705306 10.1016/j.canlet.2014.03.035

[32] Yan, Y. et al. HIF-2α promotes conversion to a stem cell phenotype and induces chemoresistance in breast cancer cells by activating Wnt and Notch pathways. *J. Exp. Clin. Cancer Res.* **37**, 256 (2018). 30340507 10.1186/s13046-018-0925-x PMC6194720

[33] Lee, P., Chandel, N. S. & Simon, M. C. Cellular adaptation to hypoxia through hypoxia inducible factors and beyond. *Nat. Rev. Mol. Cell Biol.* **21**, 268–283 (2020). 32144406 10.1038/s41580-020-0227-y PMC7222024

[34] Cai, B. et al. USP5 attenuates NLRP3 inflammasome activation by promoting autophagic degradation of NLRP3. *Autophagy* **18**, 990–1004 (2022). 34486483 10.1080/15548627.2021.1965426 PMC9196652

[35] Li, J. et al. USP5-Beclin 1 axis overrides p53-dependent senescence and drives Kras-induced tumorigenicity. *Nat. Commun.* **13**, 7799 (2022). 36528652 10.1038/s41467-022-35557-y PMC9759531

[36] Meng, J. et al. USP5 promotes epithelial-mesenchymal transition by stabilizing SLUG in hepatocellular carcinoma. *Theranostics* **9**, 573–587 (2019). 30809294 10.7150/thno.27654 PMC6376178

[37] Sun, H. et al. Stabilization of ERK-phosphorylated METTL3 by USP5 increases m6A methylation. *Mol. Cell* **80**, 633–647.e7 (2020). 33217317 10.1016/j.molcel.2020.10.026 PMC7720844

[38] Xia, P. et al. METTL5 stabilizes c-Myc by facilitating USP5 translation to reprogram glucose metabolism and promote hepatocellular carcinoma progression. *Cancer Commun.* **43**, 338–364 (2023). 10.1002/cac2.12403 PMC10009668 36602428

[39] Xiao, X. et al. ERK and USP5 govern PD-1 homeostasis via deubiquitination to modulate tumor immunotherapy. *Nat. Commun.* **14**, 2859 (2023). 37208329 10.1038/s41467-023-38605-3 PMC10199079

[40] Chen, Z. & Han, Z. C. STAT3: a critical transcription activator in angiogenesis. *Med. Res. Rev.* **28**, 185–200 (2008). 17457812 10.1002/med.20101

[41] Jia, Y., Wang, Q., Liang, M. & Huang, K. KPNA2 promotes angiogenesis by regulating STAT3 phosphorylation. *J. Transl. Med.* **20**, 627 (2022). 36578083 10.1186/s12967-022-03841-6 PMC9798605

[42] Huang, C. et al. BICC1 drives pancreatic cancer progression by inducing VEGF-independent angiogenesis. *Signal Transduct. Target. Ther.* **8**, 1–12 (2023). 37443111 10.1038/s41392-023-01478-5 PMC10344882

[43] Niu, G. et al. Constitutive Stat3 activity up-regulates VEGF expression and tumor angiogenesis. *Oncogene* **21**, 2000–2008 (2002). 11960372 10.1038/sj.onc.1205260

[44] Li, L., et al. Integrative proteogenomic characterization of early esophageal cancer. *Nat. Commun.* **14**, 1666 (2023). 36966136 10.1038/s41467-023-37440-w PMC10039899

[45] Peterson, L. F. et al. Targeting deubiquitinase activity with a novel small-molecule inhibitor as therapy for B-cell malignancies. *Blood* **125**, 3588–3597 (2015). 25814533 10.1182/blood-2014-10-605584

[46] Potu, H. et al. Usp5 links suppression of p53 and FAS levels in melanoma to the BRAF pathway. *Oncotarget* **5**, 5559–5569 (2014). 24980819 10.18632/oncotarget.2140 PMC4170643

[47] Ansari, M. J. et al. Cancer combination therapies by angiogenesis inhibitors; a comprehensive review. *Cell Commun. Signal.* **20**, 49 (2022). 35392964 10.1186/s12964-022-00838-y PMC8991477

[48] Huang, H. et al. Jag1/2 maintain esophageal homeostasis and suppress foregut tumorigenesis by restricting the basal progenitor cell pool. *Nat. Commun.* **15**, 4124 (2024). 38750026 10.1038/s41467-024-48347-5 PMC11096375

[49] Popovic, D., Vucic, D. & Dikic, I. Ubiquitination in disease pathogenesis and treatment. *Nat. Med.* **20**, 1242–1253 (2014). 25375928 10.1038/nm.3739

[50] Chu, L. et al. A phase II study of apatinib in patients with chemotherapy-refractory esophageal squamous cell carcinoma (ESO-Shanghai 11). *Oncologist* **26**, e925–e935 (2021). 33393167 10.1002/onco.13668 PMC8176978

