# Correction: Aberrant Notch-signaling promotes tumor angiogenesis in esophageal squamous-cell carcinoma

**DOI:** 10.1038/s41392-025-02403-8

**Published:** 2025-08-31

**Authors:** Cainan Li, Pujie Wu, Xiaoting Xie, Xinjie Chen, Liping Chen, Liang Zhu, Zhixuan Xuan, Tianyuan Liu, Wen Tan, Shaosen Zhang, Dongxin Lin, Chen Wu

**Affiliations:** 1 Department of Etiology and Carcinogenesis, National Cancer Center/National Clinical Research Center for Cancer/Cancer Hospital, Chinese Academy of Medical Sciences and Peking Union Medical College, Beijing 100021, China; 2 Changping Laboratory, Chinese Academy of Medical Sciences and Peking Union Medical College, Beijing 102206, China; 3 Key Laboratory of Cancer Genomic Biology, Chinese Academy of Medical Sciences and Peking Union Medical College, Beijing 100021, China; 4 Collaborative Innovation Center for Cancer Personalized Medicine, Nanjing Medical University, Nanjing 211166, China; 5 Sun Yat-sen University Cancer Center, State Key Laboratory of Oncology in South China, Guangzhou 510060, China; 6 CAMS Oxford Institute, Chinese Academy of Medical Sciences, Beijing 100006, China

**Keywords:** Tumour angiogenesis, Gastrointestinal cancer

Correction to: *Signal Transduction and Targeted Therapy* 10.1038/s41392-025-02309-5, published online 22 July 2025

We, the authors of the paper, showed that Notch-signaling activation can promote ESCC tumor angiogenesis. After online publication, we noticed, in Supplementary Fig. 6a, the H&E image of the OE ctrl group in the *USP5*-OE KYSE450 conditioned medium was partially overlapped with the OE ctrl group in the *NICD1*-OE KYSE450 conditioned medium shown in Fig. 2d of the main text. These two control groups were based on different empty vectors (GV301 and GV492) derived from the same parental KYSE450 cells. The images originated from two independent Matrigel plug assays with exactly identical experimental procedures and these two vectors are not expected to exert distinct biological effects. This correction does not affect any of the results or conclusions presented in the original publication. We sincerely apologize for any confusion this may have caused. We have re-cropped the correct image and provide it below as the updated Supplementary Fig. 6a.

Incorrected Fig. S6a
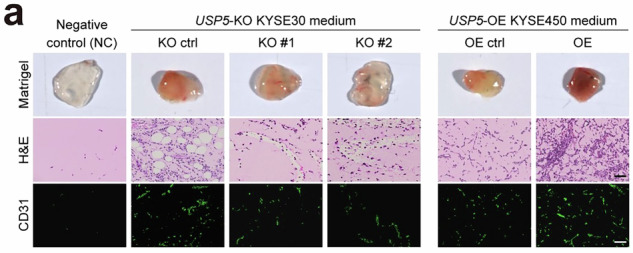


Corrected Fig. S6a
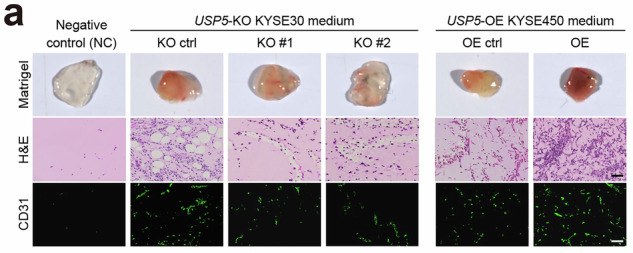


The original article has been corrected.

## Supplementary information

Correct Supplementary Fig. 6a

