# Supplementary material for: Aberrant Notch-signaling promotes tumor angiogenesis in esophageal squamous-cell carcinoma
Source: Signal Transduct Target Ther. 2025 Jul 22;10:233. doi: 10.1038/s41392-025-02309-5 (PMC12280033; doi:10.1038/s41392-025-02309-5)

**Correction: Aberrant Notch-signaling promotes tumor angiogenesis in esophageal squamous-cell carcinoma**

Correction to: Signal Transduction and Targeted Therapy (2025) 10(1):233. http://doi: 10.1038/s41392-025-02309-5, published online 22 July 2025

After online publication of the article, the authors noticed an inadvertent error in Supplementary Fig. 6a. The H&E image of the OE control group in the *USP5*-OE KYSE450 conditioned medium was mistakenly overlapped with the OE control group in the *NICD1*-OE KYSE450 conditioned medium shown in Fig. 2d of the main text. Although these two control groups were based on different empty vectors (GV301 and GV492), both were derived from the same parental KYSE450 cells and the vectors are not expected to exert distinct biological effects. The images originated from two independent Matrigel plug assays. This correction does not affect any of the results or conclusions presented in the original publication. We apologize for the oversight. We have re-cropped the correct image and provide it below as the updated Supplementary Fig. 6a.

Fig. S6a Corrected graphic figure.


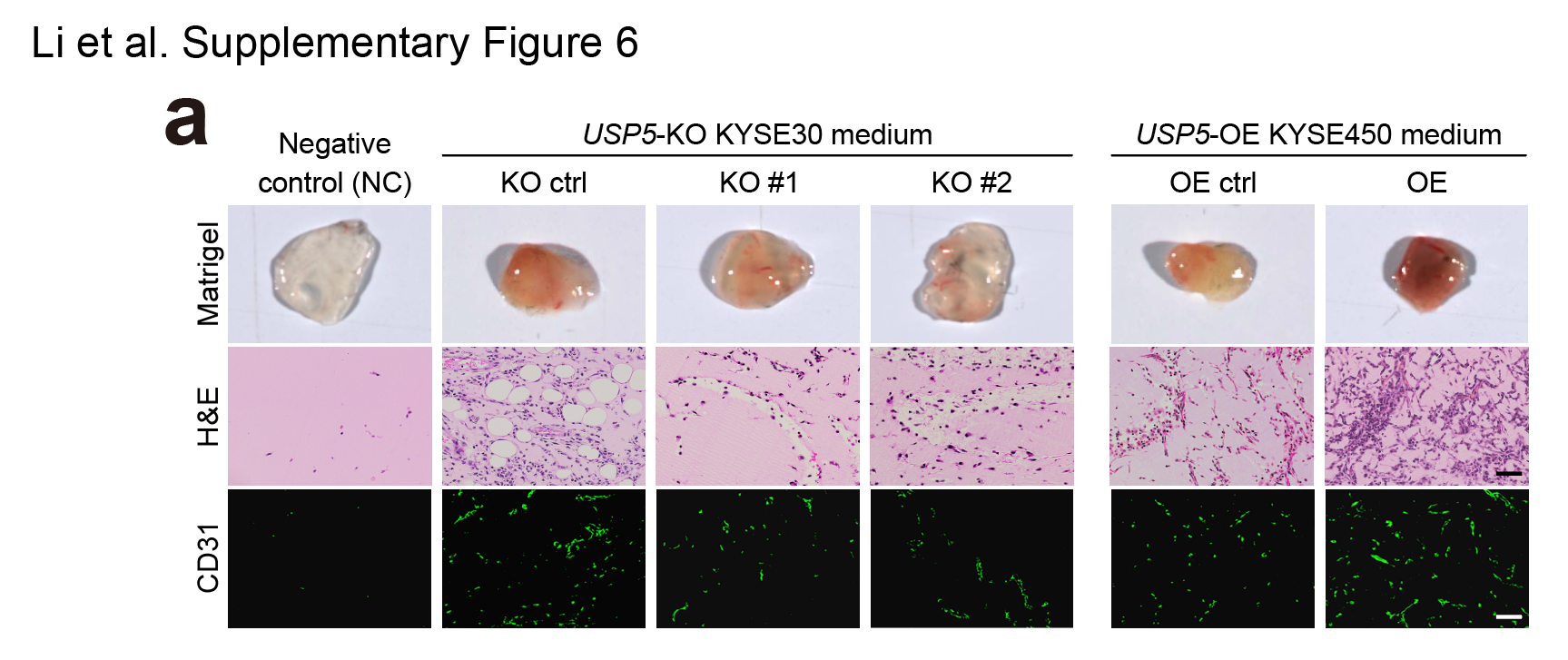

Supplement: Supplementary file 1 — Supplementary files [file 41392_2025_2309_MOESM1_ESM.docx]
