# Supplementary figures and images for: Correction: Aberrant Notch-signaling promotes tumor angiogenesis in esophageal squamous-cell carcinoma
Source: Signal Transduct Target Ther. 2025 Aug 31;10:288. doi: 10.1038/s41392-025-02403-8 (PMC12399740; doi:10.1038/s41392-025-02403-8)

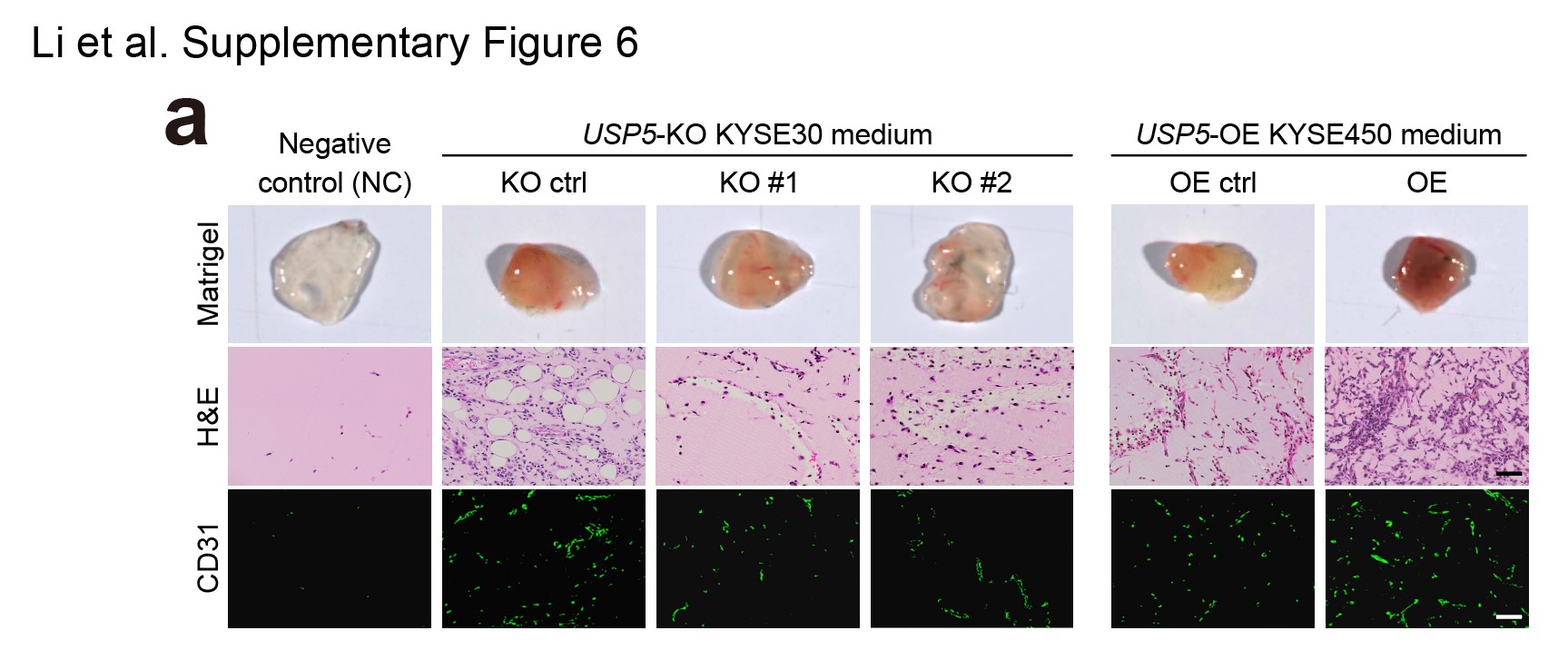

Supplement: Supplementary file 1 — Correct Supplementary Fig. 6a [file 41392_2025_2403_MOESM1_ESM.jpg]
